# Patients’, clinicians’ and the research communities’ priorities for treatment research: there is an important mismatch

**DOI:** 10.1186/s40900-015-0003-x

**Published:** 2015-06-25

**Authors:** Sally Crowe, Mark Fenton, Matthew Hall, Katherine Cowan, Iain Chalmers

**Affiliations:** 1 Crowe Associates Ltd., 15 Chinnor Road, Thame, Oxon OX9 3LW UK; 2 UK DUETs, NHS Evidence, National Institute of Health and Social Care Excellence, Level 1A, City Tower, Piccadilly Plaza, Manchester, 4BD M1 UK; 3 Institute for Evolutionary Biology, University of Edinburgh, Edinburgh, Scotland UK; 4 Katherine Cowan Consulting Ltd., 62 Marine Court, St. Leonards-on-Sea, East Sussex, TN38 0DN UK; 5 James Lind Initiative, Summertown Pavilion, Middle Way, Oxford, OX2 7LG UK

**Keywords:** Research prioritisation, James Lind Alliance, Priority setting partnerships, Research priority setting, Mismatch in research, Treatment uncertainties, UK DUETs, value in research

## Abstract

**Plain English summary:**

There is some evidence that there is a mismatch between what patients and health professionals want to see researched and the research that is actually done. The James Lind Alliance (JLA) research Priority Setting Partnerships (PSPs) were created to address this mismatch. Between 2007 and 2014, JLA partnerships of patients, carers and health professionals agreed on important treatment research questions (priorities) in a range of health conditions, such as Type 1 diabetes, eczema and stroke. We were interested in how much these JLA PSP priorities were similar to treatments undergoing evaluation and research over the same time span. We identified the treatments described in all the JLA PSP research priority lists and compared these to the treatments described in a group of research studies (randomly selected) registered publically. The priorities identified by JLA PSPs emphasised the importance of non-drug treatment research, compared to the research actually being done over the same time period, which mostly involved evaluations of drugs. These findings suggest that the research community should make greater efforts to address issues of importance to users of research, such as patients and healthcare professionals.

**Abstract:**

**Background**

Comparisons of treatment research priorities identified by patients and clinicians with research actually being done by researchers are very rare. One of the best known of these comparisons (Tallon et al. Relation between agendas of the research community and the research consumer 355:2037–40, 2000) revealed important mismatches in priorities in the assessment of treatments for osteoarthritis of the knee: researchers preferenced drug trials, patients and clinicians prioritised non-drug treatments. These findings were an important stimulus in creating the James Lind Alliance (JLA). The JLA supports research Priority Setting Partnerships (PSPs) of patients, carers and clinicians, who are actively involved in all aspects of the process, to develop shared treatment research priorities. We have compared the types of treatments (interventions) prioritised for evaluation by JLA PSPs with those being studied in samples of clinical trials being done over the same period.

**Objective**

We used treatment research priorities generated by JLA PSPs to assess whether, on average, treatments prioritised by patients and clinicians differ importantly from those being studied by researchers.

**Methods**

We identified treatments mentioned in prioritised research questions generated by the first 14 JLA PSPs. We compared these treatments with those assessed in random samples of commercial and non-commercial clinical trials recruiting in the UK over the same period, which we identified using WHO’s International Clinical Trials Registry Platform.

**Results**

We found marked differences between the proportions of different types of treatments proposed by patients, carers and clinicians and those currently being evaluated by researchers. In JLA PSPs, drugs accounted for only 18 % (23/126) of the treatments mentioned in priorities; in registered non-commercial trials, drugs accounted for 37 % (397/1069) of the treatments mentioned; and in registered commercial trials, drugs accounted for 86 % (689/798) of the treatments mentioned.

**Discussion**

Our findings confirm the mismatch first described by Tallon et al. 15 years ago. On average, drug trials are being preferenced by researchers, and non-drug treatments are preferred by patients, carers and clinicians. This general finding should be reflected in more specific assessments of the extent to which research is addressing priorities identified by the patient and clinician end users of research. It also suggests that the research culture is slow to change in regard to how important and relevant treatment research questions are identified and prioritised.

**Electronic supplementary material:**

The online version of this article (doi:10.1186/s40900-015-0003-x) contains supplementary material, which is available to authorized users.

## Background

In an important report published in 2000, Tallon and colleagues showed that patients’ and clinicians’ priorities for research on the management of osteoarthritis of the knee were not reflected in the research actually being done [1]: patients, rheumatologists, physiotherapists and general practitioners had little enthusiasm for drug trials, yet these constituted the vast majority of the published studies of treatments for this condition. Patients and clinicians both said they wanted more rigorous evaluation of the effects of physiotherapy and surgery and better assessment of the educational and coping strategies that might help patients to manage this chronic, disabling and often painful condition. A survey by Stewart and Oliver [2] suggests that this assessment of the mismatch between the research needs felt by both patients and clinicians on the one hand and current research agendas on the other appears to have been a rare, and possibly solitary, attempt to audit the extent to which research agendas are meeting the needs of the patient and clinician end users of research.

Three years after the paper by Tallon and colleagues, Chalmers and colleagues [3] compared the characteristics of randomised trials funded between 1980 and 2002 by the main non-commercial research funders in the UK. The majority of trials supported by the Medical Research Council (MRC) and the medical research charities were funded in response to proposals made by researchers, an approach called ‘responsive funding’. The majority of trials supported by the Department of Health, the NHS Research and Development Programme in England, the Chief Scientist Office in Scotland and similar sources of funding in Wales and Northern Ireland reflected priorities identified by these funders, who then called for research proposals addressing their priorities, an approach called ‘commissioned research’. The analysis demonstrated the different consequences of these two broad approaches. Trials funded by the MRC and research charities addressed a limited range of treatment types (mainly drugs) and health problems (mainly cancer and cardiovascular disease). By contrast, trials funded by the Department of Health, the NHS Research and Development Programme and similar funders in Scotland, Wales and Northern Ireland addressed many different types of treatments and a much wider range of health problems [3].

The results of the studies by Tallon et al. and Stewart and Oliver, and the consequences of the research funding processes described by Chalmers et al., were an important stimulus in creating the James Lind Alliance (JLA)—an initiative to establish and develop research Priority Setting Partnerships (PSPs) of patients, carers and clinicians to inform treatment research agendas [4]. This initiative represented a departure from existing processes used by research funders and was to some extent experimental. It was viewed by many in the research community with caution and scepticism but more positively by enough people to ensure that a wide range of partnerships were established and built on in subsequent years.

To assess whether the mismatch reported by Tallon and colleagues [1] has been reflected in the priorities identified by Priority Setting Partnerships over the first decade of the James Lind Alliance, we have compared the types of treatments prioritised by JLA PSPs with the treatments being studied in samples of clinical trials being done over the same period.

## Methods

### Identification of treatment uncertainties by JLA Priority Setting Partnerships

Treatment uncertainties are identified and prioritised by James Lind Alliance Priority Setting Partnerships (PSPs) using online and postal surveys of patients, carers and clinicians and the research literature. Surveys tend to ask open questions such as ‘What questions about X would you like to see answered by research?’ so that a wide range of people can contribute their research questions and ideas. Data are cleaned and analysed and similar uncertainties combined, usually by an information specialist, with clinical and patient input from the partnership steering group. These are checked to confirm that they reflect real uncertainties. Details of this process are published online in a JLA Guidebook [5]. An uncertainty is judged to exist when ‘no up-to-date (three years), relevant and reliable systematic reviews of research evidence addressing the uncertainty about the effects of treatment exist, or up-to-date relevant and reliable systematic reviews of research evidence show that uncertainty exists’. As part of this process of checking for genuine uncertainties, two criteria have to be met: (i) the measure of an uncertainty used by the UK Database of Uncertainties about the Effects of Treatments (DUETs) https://www.library.nhs.uk/duets/, which is when a reported confidence interval (measure of uncertainty) in a systematic review crosses the line of no effect or line of unity, and (ii) a clinician or person with relevant clinical knowledge confirms that the outcome being reported as statistically significant is also clinically relevant [6].

Uncertainties that have been resolved in up-to-date systematic reviews of research are removed from the lists of research uncertainties for prioritisation.

### Prioritisation of uncertainties by Priority Setting Partnerships

A first phase of prioritisation with the community of interest (patients, carers and clinicians) is conducted, usually online. This consists of a list of research uncertainties open for public vote, usually over 1 to 2 months. Information about voters, such as age and gender, is collected and analysed to assess the share of vote across groups. This voting process results in a shortlist of up to 30 treatment research uncertainties to be discussed at a final priority setting workshop.

Facilitators take part in a pre-workshop briefing session, stressing the underlying principles of the discussion groups and how to deploy these. A structure for discussion and voting called ‘Nominal Group Technique’ is used to arrive at the ten most important research uncertainties (priorities). Working in small groups, workshop participants discuss and rank in priority order the research uncertainties in up to three rounds, with the make-up of the small groups changing at least once. Rank scores are entered into a spreadsheet and aggregated across all small groups at each stage. The next stage of discussion and voting starts with this aggregate rank each time. After three rounds, the whole group gathers to review the final aggregate score and agree on the final ten priorities.

Participants are reminded to bear in mind their own experiences during discussion, as well as the voting patterns from the first prioritisation exercise. These discussions are often emotive and challenging. Leadership from neutral facilitators helps to ensure equitable discussion, both in terms of the amount of time that individuals use in the discussion and the interplay of professional and lay views on the uncertainties and condition(s) being considered. Discussion is typified by participants taking turns to offer views on the treatment uncertainties being discussed and presenting rationales for moving particular uncertainties up or down the rank order. Facilitators use the pressure of time to keep focus on the task and seek consensus from each group before finalising each stage of the process.

Between April 2007 and March 2013, 14 PSPs were completed using these methods:

- Asthma [7]
- Urinary incontinence [8]
- Vitiligo [9]
- Prostate cancer [10]
- Schizophrenia [11]
- Type 1 diabetes [12]
- Ear, nose and throat aspects of balance
- Life after stroke [13]
- Eczema [14]
- Tinnitus [15]
- Cleft lip and palate [16]
- Dystrophic epidermolysis bullosa [17]
- Lyme disease [18]
- Pressure ulcers

### Analysing priorities from all the JLA PSPs

The priority lists from each PSP were assembled, giving a total of 149 priorities to analyse. SC and MF classified the treatments described in these priorities, using the classification reported by Chalmers et al. [3] (Additional file 1 and Additional file 2). If two types of treatment were identified in a priority, both were entered into the analysis. Where there were more than two treatments within a priority, these were classified as ‘mixed or complex’. When no specific treatment had been identified in the priority, they were allocated to an ‘other’ category, for example, ‘Which treatments could reduce weight gain in schizophrenia?’ IC was consulted when there were classification uncertainties. One hundred and twenty-six treatments were mentioned in the 149 priorities analysed. Some PSPs prioritised more than ten questions, and some prioritised questions about issues other than treatment effects.

### Sampling concurrently registered clinical trials for comparison

The WHO International Clinical Trials Registry Platform was sampled for the comparison data. Our inclusion criteria were that studies had to be registered between January 2003 and December 2012 and that the UK was among the countries of recruitment (our search terms are provided in additional information) Additional file 3. We restricted our search to this registry as it is the most comprehensive and contains details (and links to further information) of registered studies, mostly clinical trials. Some JLA PSP priorities may be better addressed by other research methods that may not be captured in this registry. This may be a limitation of this approach and lead to under-reporting of non-drug treatment research priorities.

The contents of the records of these trials were downloaded to a spreadsheet with a script written in Python using the Beautiful Soup module (Beautiful Soup is a ‘site scraping’ tool designed to extract data from web-based documents). The list was checked for duplicate studies, which were removed. Our original sample size was 1703 research study records. After initial checking, 21 were removed as they did not meet inclusion criteria, giving a total of 1682 research studies to inspect.

A categorisation of the records into provisional ‘commercial’ and ‘non-commercial’ groups was performed by a search for keywords. This showed 52.8 % of records were non-commercial research, and the rest (47.2 %) referred to commercially funded studies.

SC and MF analysed the sampled trial citations together and agreed on the category to which each study should be assigned. Often, the authors needed to check the web links to establish exactly what the treatment was, as it was not always clear from the study title and/or there was no plain language summary available. Additional classification rules were developed for treatments that led to recurring classification questions (such as herbal preparations and food additives). Samples of 879 non-commercial trials and 803 commercial trials generated totals of 1069 and 798 mentioned treatments, respectively.

### Ethics

Ethics approval for this study was not needed as our secondary analyses had no access to any identifiable personal data.

## Results

**Fig. 1:**
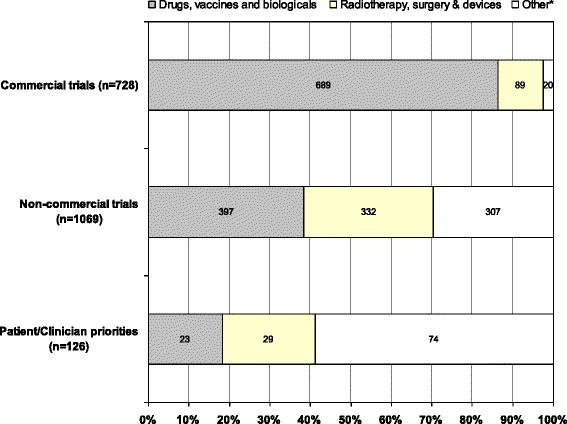
Treatments mentioned in commercial trials, non-commercial trials and research priorities identified by the James Lind Alliance Priority Setting Partnerships, 2003–2012

We found marked differences between the proportions of different types of treatments mentioned in the JLA PSP priorities and those currently being evaluated and registered on the Clinical Trials Registry. On average, in JLA PSPs, drugs accounted for 18 % (23/126) of the treatments mentioned in priorities; in registered non-commercial trials, drugs accounted for 37 % (397/1069) of the treatments mentioned; and in registered commercial trials, drugs accounted for 86 % (689/798) of the treatments mentioned (Table 1; Fig. 1).

**Table 1:** Interventions mentioned in research priorities identified by the James Lind Alliance Priority Setting Partnerships and among registered trials, 2003–2012

Type of intervention	JLA patient-clinician Priority Setting Partnerships	Registered non-commercial trials	Registered commercial trials
	Percentages (numbers) of interventions out of a total of 126 interventions mentioned	Percentage (numbers) of interventions out of a total of 1069 interventions mentioned	Percentage (numbers) of interventions out of a total of 798 interventions mentioned
Drugs, vaccines and biologicals	18.2 (23)	37.2 (397)	86.3 (689)
Radiotherapy, surgery and perioperative, devices, and diagnostic	23.0 (29)	29.8 (332)	11.1 (89)
Education and training, service delivery, psychological therapy, physical therapies, exercise, complementary therapies, social care, mixed or complex, diet, other	58.7 (74)	31.9 (307)	2.6 (20)

## Discussion

Our findings mirror those reported by Tallon and colleagues [1]: there is an important mismatch between the treatments that patients and clinicians wish to see evaluated and the treatments being evaluated by researchers. Indeed, our analysis may have underestimated the extent of the mismatch because more non-commercial than commercial trials were registered at the beginning of the time period (2003–2012) over which we aggregated data than at the end of it (data available from authors). Clearly, the research priorities identified by the James Lind Alliance Priority Setting Partnerships cannot be expected to be reflected in research being done at the same time, but the pattern we have found confirms that found by Tallon et al. and is based on a much larger sample, with data collection over a substantially longer time.

It is not a surprise that a very high proportion (86.3 %) of the registered commercial trials concerned the evaluation of drugs. However, the very low proportion (2.6 %) of registered commercial trials that studied the effects of the non-drug treatments rated important by patients and clinicians is noteworthy: it suggests that few of these drug trials can have used non-drug comparators, for example, comparing drugs with psychological therapies for treating depression.

The fact that our assessment of the research priorities of patients and clinicians has been drawn from such a wide range of health problems is a strength. Even so, it should not be assumed that similar findings would necessarily result from replications of similar analyses done for other health problems or for replications that are not limited to the very highest priorities that the James Lind Alliance Priority Setting Partnerships aim to identify.

Apart from the influence of commercial priorities, what might account for the ongoing mismatch we have shown? One obvious explanation is that the users of research evidence apparently only very rarely contribute to setting research agendas [19], so some research questions rated important by patients and clinicians may simply never occur to researchers. For example, in reference to the Asthma PSP, Professor Stephen Holgate, chair of the UK Respiratory Research Collaborative, observed that ‘Without this coming together of patients and the research community catalysed by the JLA, the subject of breathing exercises would never have been identified as one that received so much enthusiastic support.’ A randomised trial to assess the effect of breathing exercises was funded as a result [20]. There may also be ‘methodological disincentives’: designing, running and interpreting trials of drugs will usually be methodologically straightforward compared with evaluating the psychological or physical therapies, service delivery and other non-drug treatments that featured so prominently among the priorities identified by patients and clinicians.

There appear to have been few examples of researchers endeavouring to find out what questions the patient and clinician users of research would like to see addressed [21], and there are few audits of the extent to which these expressed needs have been reflected in research agendas. The research culture may be changing, as indicated in the follow-up of JLA PSP research activity [22] and other initiatives in establishing research priorities for healthcare services that fully involve patients and clinicians [23].

Follow-up has shown that some PSPs are more successful than others at sharing their research priorities and stimulating research commissioning and proposals to address their important questions. For example, the Sight Loss PSP used its priorities as a way of raising the profile and diversity of research in its area. [24] Other PSPs, such as the Life after Stroke PSP [25], have used the opportunity of a partnership to explore different methods of engagement so that a wide range of research perspectives is gathered and prioritised. Some of the research charities which participated in JLA PSPs have gone on to commission research reflecting the priorities identified.

To increase the returns on their investments, we suggest that research funders assess the extent to which the treatment research in their portfolios reflects the priorities identified by the future users and beneficiaries of the research they fund.

A recent review of public involvement by the National Institute for Health Research recommends that ‘relevance’ be one of the three measures of success of future public involvement in health and social care research [26]. We suggest that research funders, researchers and research institutions use the JLA PSP top 10 research topics, available at http://www.jla.nihr.ac.uk/top-tens.asp and UK DUETs http://www.library.nhs.uk/duets/, as a source of research questions to be developed and pursued. This will ensure that they are addressing questions that reflect the interests and needs of patients, carers and clinicians, as originally envisaged by the James Lind Alliance [27, 28].

## Conclusions

Our findings confirm the continuing mismatch first described 15 years ago by Tallon et al. [1]: research on drugs is preferred by researchers, evaluation of non-drug treatments is preferred by patients and clinicians. Our findings are relevant to research funders, research institutions and researchers themselves. If research is to reflect the priorities of patients and clinicians, leadership and incentives will be needed. The current research ‘system’ and culture is not geared to bridging the mismatch we have documented.

## Additional files

Additional file 1: **Interventions described in research priorities identified by James Lind Alliance Priority Setting Partnerships and among registered trials, 2003–2012.** Detail and numbers in each treatment category.

Additional file 2: **Specific JLA research priorities allocated to categories.** The JLA research priority questions allocated to categories.

Additional file 3: **Search terms used for the WHO International Clinical Trials Registry Platform.** The file contains the ‘non-commercial’ and ‘commercial’ keywords used.

## Abbreviations

JLA: James Lind Alliance
PSP: Priority Setting Partnership
UK DUETs: UK Database of Uncertainties about the Effects of Treatments
WHO: World Health Organization
