# Erratum to: Patients’, clinicians’ and the research communities’ priorities for treatment research: there is an important mismatch

**DOI:** 10.1186/s40900-015-0014-7

**Published:** 2015-12-23

**Authors:** Sally Crowe, Mark Fenton, Matthew Hall, Katherine Cowan, Iain Chalmers

**Affiliations:** 1 Crowe Associates Ltd., 15 Chinnor Road, Thame, Oxon OX9 3LW UK; 2 UK DUETs, NHS Evidence, National Institute of Health and Social Care Excellence, Level 1A, City Tower, Piccadilly Plaza, Manchester, 4BD M1 UK; 3 Institute for Evolutionary Biology, University of Edinburgh, Edinburgh, Scotland UK; 4 Katherine Cowan Consulting Ltd., 62 Marine Court, St. Leonards-on-Sea, East Sussex, TN38 0DN UK; 5 James Lind Initiative, Summertown Pavilion, Middle Way, Oxford, OX2 7LG UK

The authors wish to apologize to readers for an incorrect element in Table 1 and Figure 1 in the original article [1]. The correct files have been included in this erratum. We also wish to thank Sara Heesterbeek for alerting us to our error.

**Table 1:** Interventions mentioned in research priorities identified by James Lind Alliance Priority Setting Partnerships, and among registered trials, 2003-2012

Type of intervention	JLA patient-clinician Priority Setting Partnerships	Registered non-commercial trials	Registered commercial trials
	Percentages (numbers) of interventions out of a total of 126 interventions mentioned	Percentage (numbers) of interventions out of a total of 1069 interventions mentioned	Percentage (numbers) of interventions out of a total of 798 interventions mentioned
Drugs, vaccines and biologicals	18 (23)	37 (397)	86 (689)
Radiotherapy, surgery and perioperative, devices, and diagnostic	23 (29)	31 (332)	11 (89)
Education and training, service delivery, psychological therapy, physical therapies, exercise, complementary therapies, social care, mixed or complex, diet, other	59 (74)	32 (340)	3 (20)

**Fig. 1:**
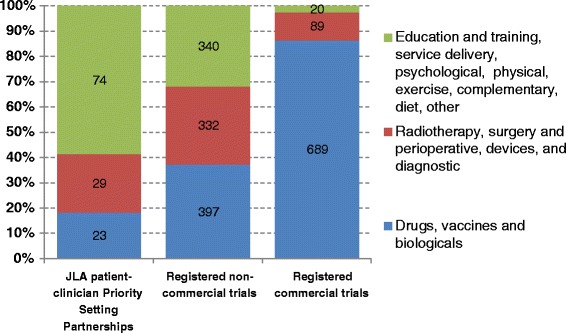
Interventions mentioned in commercial trials, non-commercial trials and research priorities identified by James Lind Alliance Priority Setting Partnerships, 2003-2012
