# Supplementary material for: Patients’, clinicians’ and the research communities’ priorities for treatment research: there is an important mismatch
Source: Res Involv Engagem. 2015 Jun 25;1:2. doi: 10.1186/s40900-015-0003-x (PMC5598091; doi:10.1186/s40900-015-0003-x)
Supplement: Supplementary file 1 — Interventions described in research priorities identified by James Lind Alliance Priority Setting Partnerships and among registered trials, 2003–2012. Detail and numbers in each treatment category. [file 40900_2015_3_MOESM1_ESM.pdf]

Additional File 1:
Interventions described in research priorities identified by James Lind Alliance Priority Setting Partnerships and among registered trials, 2003-2012

	**JLA patient-clinician Priority Setting Partnerships**<br><br>**Percentage**<br>(N out of total of 126 interventions mentioned)	**Registered non-commercial trials**<br><br>**Percentage**<br>(N out of total of 1069 interventions mentioned)	**Registered commercial trials**<br><br>**Percentage**<br>(N out of total of 798 interventions mentioned)
Drug	**15.2**<br>(19)	**34.1**<br>(364)	**83.8**<br>(669)
Radiotherapy	**0.7**<br>(1)	**1.3**<br>(14)	**0.1**<br>(1)
Surgery	**3.1**<br>(4)	**7.0**<br>(75)	**2.0**<br>(16)
Diagnostic	**10.3**<br>(13)	**8.8**<br>(94)	**2.6**<br>(21)
Education and training	**10.3**<br>(13)	**9.3**<br>(99)	**0.8**<br>(6)
Service delivery	**10.3**<br>(13)	**6.5**<br>(69)	**0.5**<br>(4)
Psychological therapy	**7.1**<br>(9)	**5.7**<br>(61)	**0.1**<br>(1)
Vaccines and biologicals	**3.1**<br>(4)	**3.1**<br>(33)	**2.5**<br>(20)
Devices	**8.7**<br>(11)	**12.7**<br>(131)	**6.4**<br>(51)
Physical therapies	**7.1**<br>(9)	**3.1**<br>(33)	**0.4**<br>(3)
Exercise	**2.3**<br>(3)	**3.1**<br>(33)	**0.0**<br>(0)
Complementary therapies	**2.3**<br>(3)	**1.1**<br>(12)	**0.0**<br>(0)
Social care	**1.5**<br>(2)	**0.0**<br>(0)	**0.0**<br>(0)
Mixed or complex	**2.3**<br>(3)	**0.0**<br>(0)	**0.0**<br>(0)
Diet	**4.7**<br>(6)	**3.1**<br>(33)	**0.8**<br>(6)
Perioperative	**0.0**<br>(0)	**1.7**<br>(18)	**0.0**<br>(0)
Other	**10.3**<br>(13)	**0.0**<br>(0)	**0.0**<br>(0)
