# Supplementary material for: Patients’, clinicians’ and the research communities’ priorities for treatment research: there is an important mismatch
Source: Res Involv Engagem. 2015 Jun 25;1:2. doi: 10.1186/s40900-015-0003-x (PMC5598091; doi:10.1186/s40900-015-0003-x)
Supplement: Supplementary file 2 — Specific JLA research priorities allocated to categories. The JLA research priority questions allocated to categories. [file 40900_2015_3_MOESM2_ESM.pdf]

Additional File 2: Specific JLA research priorities allocated to categories

Categories	James Lind Alliance Priority
**Drugs**	• What are the adverse effects associated with long term use of short and long acting bronchodilators; inhaled and oral steroids; and combination and additive therapies in children?<br>• How effective are systemic immunosuppressants in treating vitiligo?<br>• Which treatment is more effective for vitiligo: calcineurin inhibitors or steroid creams/ointments?<br>• How effective is pseudocatalase cream (combined with brief exposure to ultraviolet B) in treating vitiligo?<br>• How can sexual dysfunction due to antipsychotic-drug therapy be managed in schizophrenia? Do the adverse effects of antipsychotic drugs in schizophrenia outweigh the benefits?<br>• Which insulin's are safest and have the fewest *long term* adverse effects?<br>• What is the optimum pharmacological strategy for the management of patients with Meniere's Disease? In particular, what are the effects of betahistine (including long term effects)?<br>• Which is safer and more effective for treating eczema; steroids or calcineurin inhibitors?<br>• What is the best and safest way of using drugs that suppress the immune system? (including azathioprine, leflunomide, methotrexate, cyclosporine, mycophenolate mofetil, steroids)<br>• What is the best and safest way of using topical steroids for eczema? (Including frequency of application, potency, length of time, alternating with other topical treatments, and age limits for treatments).<br>• What is the long term safety of applying steroids to the skin?<br>• Which emollient is the most effective and safe in treating eczema?<br>• Which should be applied first emollients or topical steroids?<br>• Which medications have proven to be effective in tinnitus management compared with placebo?<br>• Should antibiotic treatment (for Lyme Disease) be continued until all symptoms have resolved? + What is the optimal course of action if symptoms persist after initial treatment (continue current treatment or change drug or dose)? + What is the optimal course of action if treatment fails i.e. if symptoms continue after the agreed course(s) of antibiotics?<br>• What is the optimal course of action (in Lyme Disease) if symptoms relapse after a treatment course is finished?<br>• How common is relapse and treatment failure (in Lyme Disease) and is it related to disease stage, gender, co-infections or any other factor?
**Radiotherapy**	• What is the effectiveness of new treatments for prostate cancer such as High Intensity Focused Ultrasound (HIFU) and Cryotherapy?
**Surgery**	• In women with prolapse (symptomatic or asymptomatic) and SUI, should suburethral tapes be inserted at the same time as repairing the prolapse?<br>• What is best practice for the management of stress urinary incontinence following failed tension free vaginal tape surgery?<br>• Is any specific surgical intervention effective in Meniere's Disease and

	what procedure is best?
**Diagnostic**	• Is urodynamic testing prior to surgery for urinary incontinence associated with better continence rates and quality of life than surgery indicated without such testing?<br>• Is there a genetic marker for prostate cancer that would be both more sensitive and more specific than PSA serum level?<br>• Are there any non-intrusive diagnostic tests that will identify aggressive prostate cancers whilst not identifying harmless cancers?<br>• Does serial PSA measurement in patients with prostate cancer accurately monitor disease progression?<br>• Does active surveillance work for treatment of prostate cancer?<br>• Would prostate cancer screening targeted at high risk groups, i.e. those with positive family history, and ethnic minorities with higher rates, improve the outcomes of treatment in these groups?<br>• How tightly controlled do fluctuations in blood glucose levels need to be to reduce the risk of developing complications in people with type 1 diabetes?<br>• What is the optimal set of guidelines for assessing children with tinnitus?<br>• Are there long-term consequences if treatment (for Lyme Disease) is delayed?<br>• Which single test and what combination of tests performs best in diagnosing or ruling out active Lyme disease, and should stage of the disease and patient age be taken into account when interpreting the tests? + How effective are the current UK tests in detecting infections due to the genospecies and strains of *B burgdorferi* sl in the UK?<br>• What key questions (clinical and epidemiological) should be considered to help make a diagnosis of Lyme disease in children and adults in the UK; would a weighting table be useful?
**Education and training**	• What is the most effective strategy to educate people with asthma and health professionals about managing the adverse effects of drug therapies?<br>• What type of patient (children and adults) and health professional education is most effective in gaining asthma control?<br>• What are the key components of successful "Self Management" for a person with asthma?<br>• Can guidance or training for general practitioners on appropriate pathways of care improve the management of patients with urinary incontinence?<br>• Do variations in GP awareness of prostate cancer affect outcomes?<br>• What training is needed to recognize the early signs of recurrence of schizophrenia?<br>• What are the characteristics of the best type 1 diabetes patient education programmes (from diagnosis to long term care) and do they improve outcomes?<br>• What makes self management successful for some people with type 1 diabetes, and not others?<br>• How can awareness of and prevention of hypoglycaemia in type 1 diabetes be improved?<br>• What is the optimal process for GP education and training for improved diagnosis and management of balance disorders?

	• What is the best way of training health professionals in the management of balance disorders?
**Service Delivery**	• What is the most effective way to manage consultations and asthma control in adolescence and young people?<br>• What is the most effective way of managing asthma with other health problems?<br>• What is the best way to treat people with schizophrenia that is unresponsive to treatment?<br>• What are the clinical, social and economic outcomes — including quality of life and the methods and effects of risk monitoring — of treatment by acute day hospitals, assertive outreach teams, in-patient units, and crisis resolution and home treatment teams?<br>• Should there be compulsory community outpatient treatment for people with severe mental disorders?<br>• What are the benefits of hospital treatment compared with home care for psychotic episodes?<br>• Does treatment of type 1 diabetics by specialists (e.g. doctors, nurses, dieticians, podiatrists, ophthalmologists and psychologists) trained in person-centred skills provide better blood glucose control, patient satisfaction and self-confidence in management of type 1 diabetes, compared to treatment by non specialists with standard skills?<br>• Which is more effective in the management of eczema; GP's, nurse-led clinics, doctor-led clinics, education programmes (How much does supporting and educating patients/carers about treatments and management of eczema help?), or multi disciplinary clinics (with dermatologist, allergy specialist, dietician)?<br>• What management strategies are more effective than a usual model of audiological care in improving outcomes for people with tinnitus?
**Psychological therapy**	• Psychological interventions for adults with asthma?<br>• How effective is the addition of psychological interventions to patients using cosmetic camouflage for improving their quality of life?<br>• How much do psychological interventions help people with vitiligo?<br>• What are the cognitive and psychological effects of living with type 1 diabetes?<br>• Are stress management techniques helpful in patients with balance disorders?<br>• What are the best ways of helping people come to terms with the long term consequences of stroke?<br>• What is the best psychological treatment for the ITCHING in eczema (e.g. habit reversal techniques)?<br>• What is the best way to improve cognition after stroke?<br>• Is Cognitive Behaviour Therapy (CBT), delivered by audiology professionals, effective for people with tinnitus? Here comparisons might be with usual audiological care or CBT delivered by a psychologist
**Vaccines and biologicals**	• What role might gene therapy play in the treatment of vitiligo?<br>• How effective are hormones or hormone-related substances that stimulate pigment cells (melanocyte-stimulating hormone analogues, afamelanotide) in treating vitiligo?<br>• Is there a vaccine that can prevent prostate cancer?

	• Does stem cell technology (e.g. for hair cell regeneration) provide an effective pipeline to develop new medical interventions for treating
**Devices**	• Are disposable catheters more or less acceptable than reusable catheters in terms of effective bladder management, patient experience and urinary tract infections?<br>• What catheter regimens are most effective in preventing urinary tract infections in patients using intermittent self-catheterisation for the management of a neurogenic bladder?<br>• Which treatment is more effective for vitiligo: light therapy or calcineurin inhibitors?<br>• How effective is ultraviolet B therapy when combined with creams or ointments in treating vitiligo?<br>• Is insulin pump therapy effective? *(immediate v deferred pump, and comparing outcomes with multiple injections)*<br>• Is it possible to constantly and accurately monitor blood sugar levels, in people with type 1 diabetes, with a discrete device (non-invasive or invasive)<br>• What type of digital hearing aid or amplification strategy provides the most effective tinnitus relief?
**Physical therapies**	• What are the benefits of breathing exercises as a form of physical therapy for asthma?<br>• What are the optimal pelvic floor muscle training protocols (frequency and duration of therapy) for the treatment of different patterns of urinary incontinence? (exercise?)<br>• What are the best interventions to improve balance/minimise symptoms in daily activities such as supermarkets, escalators etc.?<br>• What are the best treatments to improve balance, gait and mobility, including physiotherapy, gait rehabilitation, visual and auditory feedback, electrical stimulation, different types of ankle foot orthoses and electromechanical assisted gait training? (also devices – complex?)<br>• How can stroke survivors and families be helped to cope with speech problems?<br>• What is the best way to help people recover from aphasia?
**Contraception**	
**Exercise**	• Are the home-based exercises given to patients with balance disorders effective?<br>• Are exercise and fitness programmes beneficial at improving function and quality of life and avoiding subsequent stroke?
**Other (including generic treatment questions)**	• What are the most effective treatments of daytime urinary incontinence in children?<br>• Can Lyme be transmitted via other means: person to person sexually, transplacentally or by breast feeding; through organ donation; through blood transfusion?<br>• What is the best treatment for children and adults presenting with a) early Lyme disease without neurological involvement and not including erythema migrans and b) late Lyme disease of any manifestation? To include consideration of drug(s), dose, duration.<br>• What are the outcomes of cases where long term treatment (for Lyme

	Disease) has been used?<br>• Which treatment is most effective for the reduction of urinary frequency and urgency?<br>• What is the most effective way of managing asthma triggers?<br>• What can be done to delay or prevent the onset of hormone independent prostate cancer?<br>• What interventions could reduce weight gain in schizophrenia?<br>• What are the clinical benefits and cost-effectiveness of monitoring the physical health of people with schizophrenia?<br>• Is an artificial pancreas for type 1 diabetes (closed loop system) effective?<br>• What is the most effective treatment for Vestibular migraine?<br>• Are there any effective interventions for the ear pressure symptoms in Meniere's Disease?<br>• What is the best way to treat visual problems after stroke?<br>• What are the best ways to manage and/or prevent fatigue?<br>• How effective are interventions to reduce staphylococcus aureus in the management of eczema?<br>• Which is the best way to wash, including frequency of washing, water temperature, bath vs shower?<br>• How much does avoidance of irritants and allergens help people with eczema (including washing powders, soap, detergents, pollen, pets, house dust mite, carpets)?<br>• Are there different types of tinnitus and can they be explained by different mechanisms in the ear or brain?<br>• What is the link between tinnitus and hyperacusis (over-sensitivity to sounds)?<br>• What management strategies are more effective for improving tinnitus-related insomnia than a usual model of care?<br>• How can tinnitus be effectively managed in people who are Deaf or who have a profound hearing loss?
**Complimentary therapies**	• What is the role of complementary therapies in asthma management?<br>• What are the best and safest natural products to apply to the skin? (e.g. cocoa butter, aloe vera. hemp oil)<br>• Do any of the various available complementary therapies provide improved outcome for people with tinnitus compared with a usual model of care?
**Social care**	• What are the benefits of supported employment for people with schizophrenia in terms of quality of life, self esteem, long-term employment prospects and illness outcomes?<br>• What is the best way to improve confidence after stroke, including stroke clubs/groups, offering support, one-to-one input and re-skilling?
**Mixed or complex**	• What is best practice for the treatment of combined stress urinary incontinence and detrusor overactivity?<br>• How can over-treatment for prostate cancer be prevented by indentifying and excluding the treatment of harmless tumours?<br>• What are the best treatments for arm recovery and function, including visual feedback, virtual reality, bilateral training, repetitive task training, imagery/mental practice, splinting, electromechanical and robot-assisted arm training and botulinum toxin.

**Diet**	• Are there any dietary measures that can prevent prostate cancer or slow its progression?<br>• Is it helpful in preventing the severity, frequency and progression of attacks of Meniere's Disease to adopt a specific diet, or restrict salt, caffeine or fluid intake?<br>• What role might food allergy tests play in treating eczema?<br>• What is the role of diet in treating eczema? (including how effective are exclusion diets (e.g. dairy, wheat, nuts, eggs) and how effective are nutritional supplements (e.g. evening primrose, fish oils, iron, marine proteins, vitamins, fatty acids)?
**Perioperative**	
