# Supplementary material for: Patients’, clinicians’ and the research communities’ priorities for treatment research: there is an important mismatch
Source: Res Involv Engagem. 2015 Jun 25;1:2. doi: 10.1186/s40900-015-0003-x (PMC5598091; doi:10.1186/s40900-015-0003-x)
Supplement: Supplementary file 3 — Search terms used for the WHO International Clinical Trials Registry Platform. The file contains the ‘non-commercial’ and ‘commercial’ keywords used. [file 40900_2015_3_MOESM3_ESM.pdf]

Additional File 3: Search terms used for WHO International Clinical Trials Registry Platform

"Non-commercial" keywords:

NHS', 'NIHR', 'University', 'MRC', 'College', 'Research Council', 'Wellcome', 'European Commission', 'Cancer Research UK', 'Arthritis Research UK', 'Society', 'Hospital', 'Infirmary', 'Strategic Health Authority', 'BMA', 'British Medical Association', 'Department', 'Support Group', 'Institute', 'Charity', 'Trust', 'National Health Service', 'Cancer Research (UK)', 'Arthritis Research (UK)', 'Government', 'CRUK', 'Chief Scientist', 'School', 'Association', 'Agency', 'Macmillan', 'Marie Curie', 'British Heart Foundation', 'Charitable'

"Commercial" keywords:

'Corp', 'Ltd', 'GmbH', 'Novartis', 'Inc', 'Limited', 'Corporation', 's.p.a.', 'Pharma', 'ResMed', 'Actelion', 'Ipsen', 'Avita', 'Tenovus', 'Swedish Orphan Biovitrum', 'Medtronic', 'AstraZeneca', 'Company', 'Novo Nordisk', 'LLC', 'Norgine', 'Janssen Scientific Affairs', 'Abbott', 'Unilever', 'Gilead', 'Behring', 'GlaxoSmithKline', 'Bristol-Myers Squibb', 'Amgen', 'Roche', 'GSK', 'S.p.A.', 'Bayer', 'Pfizer', 'NV', 'S.A.', 'SA', 'Merck', 'INC', 'Procter and Gamble', 'LTD', 'Aventis'
